# Modified Citrus Pectin Treatment in Non-Metastatic Biochemically Relapsed Prostate Cancer: Results of a Prospective Phase II Study

**DOI:** 10.3390/nu13124295

**Published:** 2021-11-28

**Authors:** Daniel Keizman, Moshe Frenkel, Avivit Peer, Igal Kushnir, Eli Rosenbaum, David Sarid, Ilan Leibovitch, Roy Mano, Ofer Yossepowitch, David Margel, Ido Wolf, Ravit Geva, Hadas Dresler, Keren Rouvinov, Noa Rapoport, Isaac Eliaz

**Affiliations:** 1 Department of Oncology, Tel-Aviv Sourasky Medical Center, Affiliated to the Sackler School of Medicine, Tel-Aviv University, Tel-Aviv 69978, Israel; davids@tlvmc.gov.il (D.S.); idow@tlvmc.gov.il (I.W.); ravitg@tlvmc.gov.il (R.G.); 2 Department of Oncology, Rambam Medical Center, Haifa 3109601, Israel; frenkelm@netvision.net.il (M.F.); a_peer@rambam.health.gov.il (A.P.); 3 Department of Oncology, Meir Medical Center and Sackler School of Medicine, Tel-Aviv University, Kfar-Saba 4428164, Israel; igi_ku@yahoo.com (I.K.); noa.rapoport@clalit.org.il (N.R.); 4 Department of Oncology, Rabin Medical Center, Petah-Tikva 4941492, Israel; eliros@clalit.org.il; 5 Department of Urology, Meir Medical Center, Kfar-Saba 4439246, Israel; leibovitchi@clalit.org.il; 6 Department of Urology, Tel-Aviv Sourasky Medical Center, Tel-Aviv 69978, Israel; roymano78@gmail.com (R.M.); ofery@tlvmc.gov.il (O.Y.); 7 Department of Urology, Rabin Medical Center, Petah-Tikva 4941492, Israel; sdmargel@gmail.com; 8 Department of Oncology, Shaare Zedek Medical Center, Jerusalem 9103102, Israel; hadasdr@gmail.com; 9 Department of Oncology, Soroka Medical Center, Beer-Sheva 8428760, Israel; kerenruv@gmail.com; 10 Amitabha Medical Clinic and Healing Center, Santa Rosa, CA 95401, USA; isaac.eliaz@gmail.com

**Keywords:** modified citrus pectin, non-metastatic biochemically relapsed prostate cancer, PSA doubling time, PectaSol

## Abstract

Optimal therapy of biochemically relapsed prostate cancer (BRPC) after local treatment is elusive. An established modified citrus pectin (PectaSol^®^, P-MCP), a dietary polysaccharide, is an established antagonist of galectin-3, a carbohydrate-binding protein involved in cancer pathogenesis. Based on PSA dynamics, we report on the safety and the primary outcome analysis of a prospective phase II study of P-MCP in non-metastatic BRPC based. Sixty patients were enrolled, and one patient withdrew after a month. Patients (*n* = 59) were given P-MCP, 4.8 grams X 3/day, for six months. The primary endpoint was the rate without PSA progression and improved PSA doubling time (PSADT). Secondary endpoints were the rate without radiologic progression and toxicity. Patients that did not progress by PSA and radiologically at six months continued for an additional twelve months. After six months, 78% (*n* = 46) responded to therapy, with a decreased/stable PSA in 58% (*n* = 34), or improvement of PSADT in 75% (*n* = 44), and with negative scans, and entered the second twelve months treatment phase. Median PSADT improved significantly (*p* = 0.003). Disease progression during the first 6 months was noted in only 22% (*n* = 13), with PSA progression in 17% (*n* = 10), and PSA and radiologic progression in 5% *(n* = 3). No patients developed grade 3 or 4 toxicity.

## 1. Introduction

With over 207,000 newly diagnosed cases in the U.S. annually, prostate cancer is the second most widespread cancer in men in the U.S. [1]. While localized treatment modalities often cure patients with localized disease, approximately 30% demonstrate biochemical relapse at ten years. The ideal management of these patients remains elusive at present. Radiation treatment given for suspected recurrent malignant disease after a period of observation after prostatectomy, also known as salvage radiation therapy (SRT), provides long-term benefits in some patients, and the use of androgen deprivation treatment (ADT) continues to be debatable [2]. While ADT effectively reduces serum PSA levels in the majority of patients, its long-term benefits on survival and quality of life remain unclear. Data emphasize the incidence of accumulative toxicities with ADT, which may offset any potential survival benefit from early intervention and impact the life quality [3].

The natural history of men with biochemically relapsed, non-castrate prostate cancer is quite mixed. They may remain asymptomatic and free of clinical evidence of disease for many years [4]. Extensive data on patients’ natural history of relapsing after surgery and after curative radiotherapy indicate that the PSA doubling time (PSADT) predicts the probability of metastasis-free and prostate cancer-specific survival [5,6,7,8,9,10,11]. PSADT of <3 months, 3.00-8.99 months, and ≥nine months may be associated with poor, intermediate, and good prognosis in disease progression and development of overt metastatic disease. Furthermore, the Prostate-Specific Antigen Working Group’s guidelines on PSADT determined that clinical evidence supports PSADT as a predictive marker of cancer progression among post-local therapy prostate cancer patients experiencing biochemical recurrence [12]. Thus, PSADT has been used in our study design to define endpoints.

Evaluating new compounds in this patient population remains a challenge because of the lack of validated methodology. The time required until conventional clinical and radiological endpoints occur is often lengthy. Knowing PSADT and other dynamics of PSA levels in predicting the outcome of this population, changes in doubling time observed during treatment have been a popular approach applied in clinical studies designed for screening potentially active compounds [4,13,14].

PectaSol^®^ Modified Citrus Pectin (P-MCP; ecoNugenics Inc, Santa Rosa, CA, USA) is derived from the pith of citrus fruit peels and treated with enzymes, pH, and temperature. It is an orally administered competitive inhibitor of galectin-3 (Gal-3), a carbohydrate-binding protein involved in cancer pathogenesis. P-MCP is a dietary supplement form of pectin comprised of low-molecular-weight and low degree of esterification to allow absorption from the small intestinal epithelium into the circulation. Untreated pectin fibers are too long and large in structure. Thus, they are indigestible dietary fibers that pass through the gastrointestinal tract. Pectin is classified by the US-FDA as generally regarded as safe (GRAS). P-MCP produces pleiotropic effects, including but not limited to its antagonism of Gal-3, which have shown benefit in preclinical and clinical studies. Preclinical and clinical data suggest that P-MCP is active in prostate cancer patients [15,16,17]. Specifically, in cancer, P-MCP modulates several rate-limiting steps of the metastatic cascade [18]. P-MCP can also affect cancer cell resistance to chemotherapy and sensitivity to radiation. In addition, studied in fibrotic diseases, P-MCP modulates many of the steps involved in the pathogenesis of organ fibrosis and reduces fibrosis to the kidney, heart, liver, and adipose tissue. Other benefits of MCP include detoxification, anti-inflammatory, antioxidant, and improved immune function [18].

The exceptionally low incidence of toxicities and possible clinical effect of P-MCP supports further testing of this compound. In addition, P-MCP’s mechanisms of action would suggest that this compound may be particularly interesting for delaying progression in a group of patients with relatively low disease burden states, such as in the non-metastatic biochemically relapsed paradigm. To evaluate P-MCP’s clinical activity in this early disease state, we employed previously reported methodology [4,13,14] for appraising the safety and preliminary efficacy of non-hormonal compounds on the progression of relapsed, non-metastatic prostate cancer patients. The results (primary outcome analysis) of our prospective phase II study are reported.

## 2. Materials and Methods

### 2.1. Inclusion Criteria 

Eligible patients were ≥21 years old and had histologically proven prostatic adenocarcinoma. All participants had undergone radical prostatectomy and/or external beam radiation therapy, or brachytherapy, with, subsequently, a confirmed rising serum PSA level (in at least three consecutive tests, at least two weeks apart) of ≥0.2 ng/mL after radical prostatectomy or ≥2 ng/mL above nadir after radiation therapy. Patients’ participation required no evidence of locoregional or distant metastasis determined by a positron emission tomography (PET) prostate-specific membrane antigen (PSMA) scan. All previous local treatment modalities, including radiation and surgery, were completed at least three months before treatment in this study. Patients with prior systemic treatment with androgen deprivation therapy (ADT), experimental drugs, high-dose steroids, or other cancer treatments were discontinued at least six months before study admission. All patients had a normal level of serum testosterone > 150 ng/mL, and adequate bone marrow (absolute neutrophil count ≥ 1.5 × 10^3^/L, platelet count ≥ 100 × 10^3^/L), renal (creatinine ≤ 2.5 times the normal upper serum limit), and liver (total bilirubin ≤ 1.5 mg/dL, aspartate aminotransferase (AST) and alanine transaminase (ALT) ≤ 2 × upper limit of normal range) functions. In addition, all patients had an Eastern Cooperative Oncology Group (ECOG) performance status ≤ 2, a life expectancy > 6 months at study entry. Men were excluded if they had an uncontrolled intercurrent illness that limited study compliance. All participating patients signed an institutional review board (IRB)-approved consent form. Clinical Registry; NCT01681823; https://clinicaltrials.gov/ct2/show/NCT01681823, accessed on 22 November 2021.

**Figure 1:**
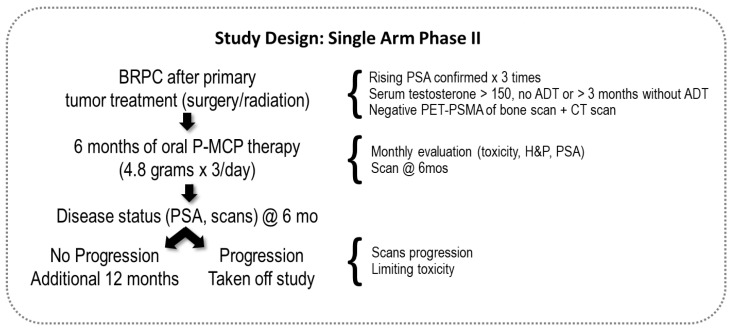
Study design. BRPC (Biochemically Relapsed Prostate Cancer); P-MCP (PectaSol^®^ Modified Citrus Pectin); @ 6mo (at six months); ADT (Androgen Deprivation Treatment); PET-PSMA (Positron Emission Tomography-Prostate-Specific Membrane Antigen Scan; CT (Computed Tomography Scan); H&P (History and Physical).

Study participants were recruited from five medical centers in Israel (Meir, Rabin, Rambam, Soroka, and Tel-Aviv Sourasky). The sponsor provided P-MCP (PectaSol-C^®^, EcoNugenics, Inc., Santa Rosa, CA, USA) to be orally taken at 4.8 grams × 3/day given to patients in packs of 270 capsules. An illustration of the study design is in Figure 1.

### 2.2. Toxicity and Disease Status at Follow-Up Monthly Assessments 

Patients were evaluated for toxicities, physical exams, serum PSA, testosterone, CRP, and galectin-3 levels. Complete assessments of disease status included positron emission tomography PET–PSMA scan after six months in patients without clinical or PSA progression or earlier upon clinical or PSA progression.

### 2.3. Treatment Duration

The treatment continued until biochemical or clinical disease progression or dose-limiting toxicity. Biochemical progression was characterized as a ≥25% increase of PSA level at six months over the baseline, without a PSADT prolongation. Clinical disease progression is defined as any new monthly evidence of progression upon a digital rectal examination or scans at six months, suggestive of local or distant disease recurrence. The duration time was defined as the time from treatment initiation to the first observation of a termination event, death by any cause, or discontinuation of treatment for any reason. Patients without evidence of disease progression (PSA and/or radiologically) or dose-limiting toxicity after six months were given an additional twelve months of treatment.

### 2.4. Safety Evaluation of Toxicity

Toxicity was defined according to the NCI Common Toxicity Criteria. Treatment would be stopped for a patient with grade 3–4 toxicity. At that point, patients would be followed weekly until ≤grade 1 and then reinitiate treatment. Therapy would be stopped upon the recurrence of grade 3/4 events and for any toxicity requiring more than four weeks to recover to ≤grade 1.

### 2.5. Statistical Analysis

The analysis was performed using the first six months of administration with the investigational supplement. This study’s primary efficacy endpoint was the rate of patients without PSA progression (defined as an increase of ≥25% from baseline) and/or patients with improvement (lengthening) of PSADT versus baseline. PSADT calculation used the natural log of two divided by the slope found from measuring a linear regression with the natural log of PSA against time (months). All the available PSA measurements in the year before patient enrollment were used to calculate baseline pre-treatment PSADT. The post-baseline PSADT was calculated using PSA levels obtained at baseline and monthly during treatment. Secondary endpoints were the rate of patients without radiologic progression, toxicity, and treatment benefits according to the PSADT risk grouping (e.g., poor < 3 months, intermediate 3–8.99 months, and good ≥ nine months).

A cohort size of sixty patients provided 85% statistical power was used to evaluate the decrease in PSA progression rate at six months, from 80% (reported data about the natural history of PSA dynamics without active therapy) [4] to 40% (with P-MCP therapy), and PSADT improvement (lengthening) rate from 25% (reported natural history without active treatment) [19] to 50% (with P-MCP therapy).

Comparisons between pre-and post-treatment parameters and within groups were analyzed by Wilcoxon Signed Rank for abnormally distributed data or a two-tailed Student t-test for normally distributed data, with results reported as a number, percentage, mean or median, and standard deviation (SD). 

## 3. Results

### 3.1. Patients

Seventy-five (*n* = 75) patients were assessed for inclusion. Fifteen patients were considered to be out of the inclusion criteria. The reasons for exclusion were metastatic disease (*n* = 13) and lack of a confirmed rise in PSA (*n* = 2). Thus, sixty eligible patients (median age 74 years, range 53–89 years) were included. The primary tumor treatment consisted of surgery in 13% (*n* = 8), radiation in 57% (*n* = 34), and both in 30% (*n* = 18). Patients with PSA progression by the present study criteria (i.e., an increase of ≥25% from baseline) within the six months before treatment initiation was noted in 88% (*n* = 53). The characteristics of the patients is in Table 1.

### 3.2. Toxicity and Compliance

**Figure 2:**
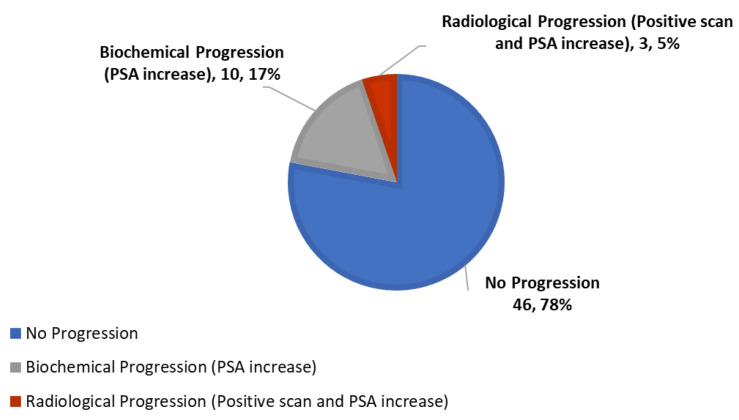
Response to therapy, no progression defined as a decreased/stable PSA and/or improvement of PSADT (*n*, %).

No patients with severe grade 3/4 toxicity were reported. Twenty percent (*n* = 12) had grade 1 toxicity (bloating) that was transient and reversible and did not require treatment discontinuation. One patient (with a baseline intermediate-risk PSADT) withdrew consent after one month. Of the remaining 59 patients, after six months, 78% (*n* = 46) responded to therapy with a decrease or stabilization of PSA, and/or improvement (lengthening) of PSADT, and with negative scans, and entered the second 12 months treatment phase. Specifically, versus baseline pre-treatment, 75% (*n* = 44) had improvement (lengthening) of PSADT, and 58% (*n* = 34) had a stabilization/decrease of PSA. Median PSADT improved significantly (*p* = 0.003), with a median (range) pretreatment PSADT of 9.12 (1.4–55) months versus a median (range) post-treatment PSADT of 15.2 (1.4–677.0) months. Table 2 and Figure 2 summarize treatment characteristics and response for prostate-specific antigen level, doubling time changes, and disease progression. 

### 3.3. Analysis of the PSADT

**Figure 3:**
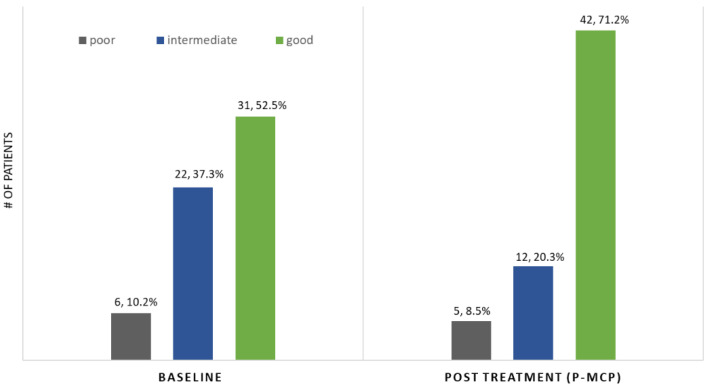
PSADT risk grouping: pre- versus post-P-MCP (*n*, %).

The benefits of therapy in terms of PSA stabilization (no change in level)/decrease and/or PSADT lengthening (% of patients and median) were seen in all PSADT risk groups (Table 2). In addition, there was a favorable change of the PSADT risk grouping during therapy (Table 2, Figure 3), with a decrease in the number of patients with a poor (<3 months) and intermediate PSADT (3.00–8.99 months) risk from 48% (*n* = 29) before therapy to 29% (*n* = 17) after therapy and an increase of the number of patients with a good risk PSADT (≥9.00) from 52% (*n* = 31) before therapy to 71% (*n* = 42) after therapy. 

**Figure 4:**
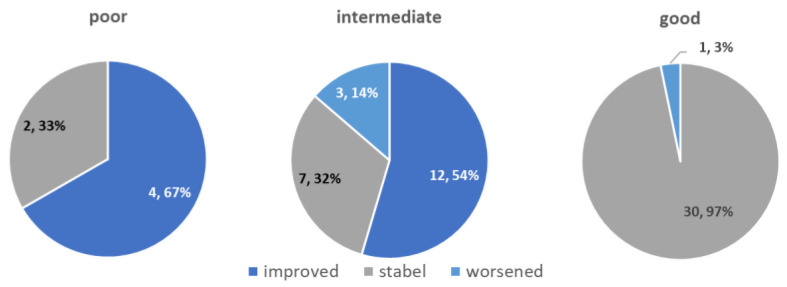
Post-P-MCP treatment change of PSADT in different pre-treatment PSADT risk groups (*n*, %).

Overall, 27% of patients (*n* = 16) favorably changed their PSADT risk grouping. Specifically, 55% (*n* = 12/22) of patients with baseline intermediate PSADT risk grouping (3–8.99 months) improved it to a good risk (≥nine months), and 66% (*n* = 4/6) of patients with baseline poor PSADT risk grouping (<3 months) improved it to intermediate risk (Figure 4).

A subgroup analysis of the PSADT risk grouping (Table 1 and Table 2) revealed a significant change of median PSADT after P-MCP therapy (versus baseline) in patients with good (median PSADT 20.4 versus 14.7 months, *p* = 0.0006) and intermediate (6.5 versus 5.21 months, *p* = 0.0025) risk.

Disease progression during the first six months of therapy was noted in only 22% (*n* = 13), with PSA progression (an increase of ≥25% from baseline) only (without radiographic progression) in 17% (*n* = 10), and both PSA and radiological progression in 5% (*n* = 3). Of note, in all, three patients with a radiographic progression, on-treatment PSA progression (an increase of ≥25% from baseline), and no lengthening of PSADT were observed.

## 4. Discussion

This clinical trial evaluated the feasibility, safety, and benefit of P-MCP in prostate cancer patients with increasing PSA levels following radiation or radical prostatectomy. The study met its primary objective, with 58% of patients without PSA progression and 75% with lengthening of PSADT versus baseline. Furthermore, the rates of patients without PSA progression and with PSADT lengthening were observed, regardless of the baseline PSADT risk grouping. Again, under P-MCP therapy, most patients with a baseline poor or intermediate risk PSADT improved their PSADT risk grouping (i.e., changed from poor to intermediate and intermediate to good). In addition, only 5% of patients had a meaningful disease progression (radiological) under therapy. Finally, a subgroup analysis of the PSADT risk groups revealed a significant change of PSADT (post-P-MCP therapy versus baseline) in patients with intermediate and good risk. At present, such an effect in the small (*n* = 6) poor-risk group could be observed, and further studies with a more substantial number of men with poor-risk PSADT are needed to confirm such an effect.

Since the eligible patients have no other evidence of active disease at the time of enrollment, according to previously reported clinical trials in such patients [4,14], we relied on PSA dynamics changes as a potential signal for antitumor activity. Based on prior data, any expression of PSA dynamics (e.g., PSADT, PSA slopes) represents the strongest prognosticator in this population [4,11]. Although the number is small, and no definitive conclusions can be drawn, in all three patients with on-treatment radiological disease progression in the present study, on treatment PSA progression and no lengthening of PSADT were observed (in accordance with data suggesting that the endpoints of PSA dynamics and PSADT are in correlation with disease control).

The sample size was computed to identify a 50% decrease in the rate of disease progression observed at six months, from 80% to 40%. This endpoint was selected based on preceding data in this patient cohort, indicating that 80% of the eligible patients for this study would continue to demonstrate evidence of progression at 6-months without treatment [4]. While the 50% decrease in progression rate at 6-months has not been validated in relation to clinically relevant events (for example, bone metastasis or survival), it was used as an endpoint in previous studies in this patient population [4]. Therefore, we feel that this represents a realistic choice of potential clinical significance to employ in initial screening for a signal of activity. Additional validation of this approach will require specially designed phase III trials.

The incidence and severity of adverse drug-related reactions were modest and reversible, and most patients stayed on treatment per protocol. These findings are consistent with the FDA classification of P-MCP as GRAS and support that this compound is appropriate for long-term treatment.

The observations of PSA stabilization/response and lengthening of PSADT in patients treated with P-MCP are consistent with prior preclinical and clinical data. The extracellular galectin-3 protein participates in the tumorigenesis process by various mechanisms, including inflammation, cellular proliferation, angiogenesis, and progression to an overt metastatic state via cancer cell–endothelial adhesion in distant organs. Elevated galectin-3 serum level was reported in prostate cancer patients [16,17,18,19,20,21,22,23,24,25,26]. P-MCP is an oral competitive inhibitor of galectin-3, and preliminary preclinical and clinical data suggest that it is active in patients with prostate cancer [17,18].

The major limitation of our study is the lack of a placebo arm. A placebo arm was considered; however, given the perceived positivity of P-MCP, a placebo control was felt to pose difficulties for patient accrual. Furthermore, although retrospective studies have shown that PSADT is a strong predictor of metastasis-free survival [11] and overall survival [7,9], or both [19,27], another limitation is whether changes in PSA and PSADT are acceptable endpoints for clinical trials in this patient population.

## 5. Conclusions

The present study suggests that P-MCP in BRPC has a potential benefit and is safe, as evident by changes in PSADT, lower than expected rates of disease progression compared to historical data, and no significant toxicity. The exceptionally low incidence of toxicities and possible clinical activity of P-MCP observed in the present study supports further testing of this compound in this patient population. Furthermore, P-MCP’s mechanisms of action suggest that this compound may be particularly interesting for delaying disease progression in a group of patients with relatively low disease burden states, such as in the non-metastatic biochemically relapsed prostate cancer. For more definitive conclusions regarding the efficacy of P-MCP in this patient population, further testing in prospective randomized studies evaluating more conventional disease endpoints is warranted.

## Figures and Tables

**Table 1:** Summary of the pre-treatment patient characteristics.

Parameter	Pre-Treatment (*n* = 60)
Age (years): Median (range)	73 (53–89)
Gleason: % (*n*)	
6	30% (*n* = 18)
7	47% (*n* = 28)
8–10	23% (*n* = 14)
Local therapy: % (*n*)	
Radical prostatectomy	13% (*n* = 8)
Radiation therapy (RT)	57% (*n* = 34)
Surgery + RT	30% (*n* = 18)
Prior ADT	52% (*n* = 31)
PSA (ng/mL): Median (range)	4.13 (0.25–30)
PSA progression (increase of ≥25% within the six months prior to treatment initiation)	88% (*n* = 53)
PSADT (months) risk grouping: % (*n*)	
Poor < 3	10% (*n* = 6)
Intermediate 3–8.99	38% (*n* = 23)
Good ≥ 9	52% (*n* = 31)
PSADT (months): Median (range)	
Whole cohort	9.12 (1.4–55)
Poor PSADT risk	2.3 (1.6–2.82)
Intermediate risk	5.21 (3.23–8.94)
Good risk	14.74 (9.10–54.6)

**Table 2:** Treatment characteristics and response at 6 months.

Parameter	Whole Cohort (*n* = 59)	According to Pre-Study PSADT (months) Risk Grouping		
		Poor <3.00 (*n* = 6)	Intermediate 3.00–8.99 (*n* = 22)	Good ≥9.00 (*n* = 31)
Overall response to therapy (Decrease or stabilization of PSA, and/or lengthening of PSADT, with negative scans)	78% (*n* = 46)	66% (*n* = 4)	77% (*n* = 17)	81% (*n* = 25)
PSA response				
Stable–decreased	58% (*n* = 34)	0% (*n* = 0)	45% (*n* = 10)	77% (*n* = 24)
Progression	42% (*n* = 25)	100% (*n* = 6)	55% (*n* = 12)	23% (*n* = 7)
PSADT months (median range)	15.3 (1.4–677)	2.35 (1.4–2.97)	6.5 (3.2–8.1)	20.4 (9.2–677)
PSADT risk grouping		9% (*n* = 5)	20% (*n* = 12)	71% (*n* = 42)
PSADT lengthening	75% (*n* = 44)	66% (*n* = 4)	82% (*n* = 18)	71% (*n* = 22)
Better PSADT risk grouping	27% (*n* = 16)	66% (*n* = 4)	55% (*n* = 12)	not applicable
Radiologic response				
Negative scans	95% (*n* = 56)	83% (*n* = 5)	91% (*n* = 20)	100% (*n* = 31)
Disease progression	5% (*n* = 3)	17% (*n* = 1)	9% (*n* = 2)	0% (*n* = 0)

## Data Availability

Provided upon request.
